# Prevalence of Sarcopenia and Its Association With Diabetes: A Meta-Analysis of Community-Dwelling Asian Population

**DOI:** 10.3389/fmed.2021.681232

**Published:** 2021-05-20

**Authors:** Seung Min Chung, Jun Sung Moon, Min Cheol Chang

**Affiliations:** ^1^Division of Endocrinology and Metabolism, Department of Internal Medicine, Yeungnam University, Daegu, South Korea; ^2^Department of Physical Medicine and Rehabilitation, College of Medicine, Yeungnam University, Daegu, South Korea

**Keywords:** diabetes mellitus, type 2, geriatrics, sarcopenia, prevalence, meta-analysis

## Abstract

**Purpose:** Sarcopenia is a major disease affecting mortality and quality of life in the elderly population. We performed a meta-analysis of studies on the community-dwelling population to investigate the prevalence of sarcopenia and its association with diabetes.

**Methods:** Databases were searched for studies published up to February 3, 2021, reporting the prevalence of sarcopenia in patients with and without diabetes. Data extraction and quality assessment were performed according to the Newcastle-Ottawa scale.

**Results:** Six articles were included in the systematic review. All the patients were Asian, aged ≥60 years (women 53.4%), and the diabetic and non-diabetic population was 1,537 and 5,485, respectively. In all six studies, the Asian Working Group for Sarcopenia criteria were used to diagnose sarcopenia. The prevalence of sarcopenia was 15.9% in diabetics and 10.8% in non-diabetics. Diabetics showed a significantly higher risk of sarcopenia than non-diabetics (pooled OR = 1.518, 95% CI = 1.110 to 2.076, Z-value = 2.611, *p* = 0.009).

**Conclusion:** Among the Asian community-dwelling geriatric population, the prevalence of sarcopenia was significantly higher in diabetics than in non-diabetics. These results suggest that strategies for the management of sarcopenia are required in Asian elderly patients, especially with diabetes.

## Introduction

Globally, rapid increase in the aging population has led to social and health issues associated with age-related chronic diseases. Sarcopenia is a syndrome of age-related degenerative skeletal disease characterized by a progressive and generalized reduction in muscular mass, strength, and function (1). Although controversies remain regarding definitions, assessment tools, diagnostic criteria, and treatment, sarcopenia is developing into a critical public health burden among the elderly, being related to adverse outcomes such as frailty, disability, poor quality of life, and increased mortality. Previous meta-analyses have reported the prevalence of sarcopenia in community-dwelling older adults, and the range varied from 9.9 to 40.4% depending on the definition of sarcopenia used (2, 3). Whereas, the earlier criterion was based only on muscle mass assessment (4), the recent definitions (5–7) include ethnic differences as well as muscle strength and function, which are more strongly associated with outcomes such as mortality (8).

The incidence of type 2 diabetes increases with age, and recent studies have revealed that both sarcopenia and diabetes have a bidirectional relationship (9). Age-related skeletal muscle degeneration could deteriorate insulin sensitivity and lead to the development of metabolic disorders such as diabetes, and vice versa. Diabetes-related decline in muscle mass, strength, and function leads to sarcopenia and increased disability, fracture, and mortality (10–12).

Although the decline of muscle mass and muscle strength has been compared between diabetic and non-diabetic patients in previous studies (13–16), few studies have compared diabetic and non-diabetic patients using the standardized sarcopenia diagnostic criteria reflecting qualitative changes in the skeletal muscles. To evaluate the prevalence of sarcopenia in diabetics and to compare it to that in non-diabetics, a meta-analysis of studies on the community-dwelling population using standardized criteria is required. Therefore, we systematically reviewed the literature and performed a meta-analysis to evaluate the impact of diabetes on the occurrence of sarcopenia in geriatric adults.

## Methods

### Search Strategy

This meta-analysis was conducted in accordance with the Preferred Reporting Items for Systematic Reviews and Meta-Analysis guidelines. We systematically searched for relevant articles in the PubMed, Embase, Cochrane Library, SCOPUS, KoreaMed, Wangfang, and Ichushi databases for studies published from January 01, 1979 to February 03, 2021. The following keywords were used in the search: (sarcopenia OR muscle mass OR muscle strength) AND (prevalence OR epidemiology OR frequency OR incidence) AND (diabetes).

### Study Selection

We applied the following inclusion criteria for the selection of articles: (1) the prevalence rate of sarcopenia was evaluated in the community-dwelling general population, (2) the prevalence rate of sarcopenia was investigated in diabetic and non-diabetic populations, (3) the age of included subjects was ≥60 years, and (4) loss of appendicular muscle mass and functional decline were considered for the diagnosis of sarcopenia. The exclusion criteria were as follows: (1) repeat publications and (2) lack of reporting on study outcomes.

### Data Extraction

After discarding duplicate studies, two reviewers (SC and MC) independently evaluated the potentially eligible studies. The articles were screened for eligibility based on a review of the title and abstract, and disagreements were resolved through consensus. After screening, the full texts of the eligible articles were read independently by the two reviewers, and the eligibility of each article was re-assessed. Subsequently, the data, including the first author, publication date, study type, number of patients, and demographic information (age, sex, and other participant details) were extracted.

### Quality Assessment

The Newcastle-Ottawa scale (NOS) for cross-sectional study was used for quality assessment (17), considering the three aspects: selection of subjects, comparability of groups, and assessment of outcome. The NOS allows four stars for selection of subjects, two for comparability of groups, and three for assessment of outcomes, a maximum of nine stars in total. Judgment of bias was expressed as “unsatisfactory studies,” “satisfactory studies,” “good studies,” or “very good studies.” The quality of each study was graded as low (0–3), moderate (4–6), or high (7–9). All divergences were resolved by consensus.

### Grading of Evidence

The overall quality (certainty) of evidence was assessed using the GRADE system which grades evidence as high, moderate, low, or very low quality. Randomized controlled trials are graded as high-quality evidence by default. Scores can then be downgraded on the basis of the following prespecified criteria: risk of bias (weight of studies shows important risk of bias), inconsistency (substantial unexplained interstudy heterogeneity), indirectness (present of factors that limit the generalizability of the results), imprecision (95% confidence interval [CI] for risk estimates are wide), and publication bias (evidence of small-study effects) (18).

### Statistical Analyses

The extracted data were analyzed statistically using Comprehensive Meta-analysis Version 2.0 (Biostat Inc., Englewood, New Jersey). For each analysis, a heterogeneity test was conducted using the I^2^ statistic, which helps to evaluate the extent of inconsistency among the results. If I^2^ values were ≥50%, the data were considered to have substantial heterogeneity, and the random-effects model was used for data analysis. In contrast, if I^2^ values were <50%, the pooled data were considered homogenous, and the fixed-effects model was applied for data analysis.

The pooled prevalence and 95% CI for sarcopenia were also calculated. Additionally, to analyze the difference in the risk of sarcopenia between diabetes and non-diabetes, we analyzed the odds ratio (OR), and 95% CIs from the raw prevalence data. Statistical significance was set at *P* < 0.05.

## Results

### Study Selection

**Figure 1:**
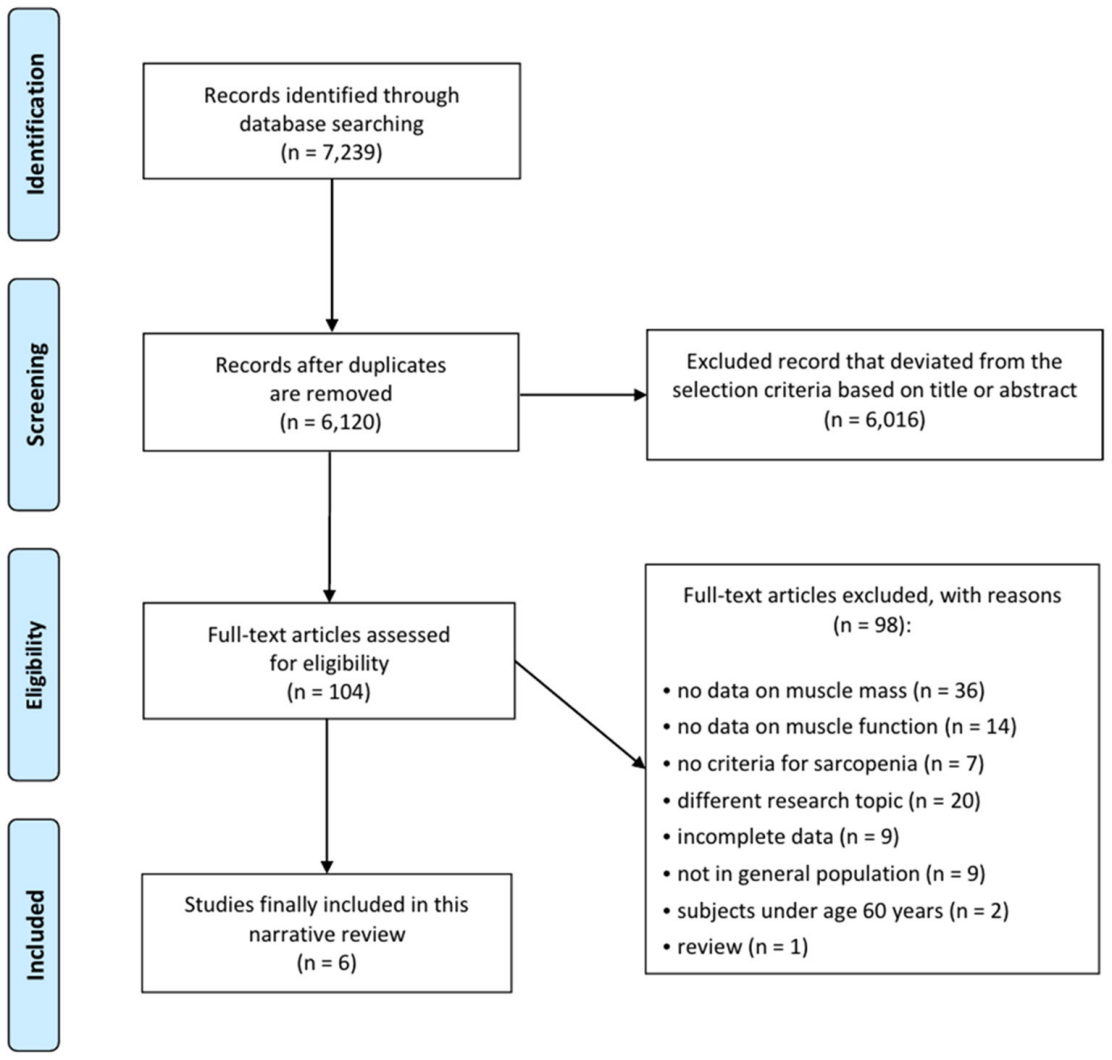
Flow chart depicting the search results of the meta-analysis.

A total of 7,239 potentially relevant studies were selected for the preliminary search from all databases (Figure 1). We initially excluded 1,119 duplicate studies, and excluded an additional 6,016 publications after reviewing their titles and abstracts. The remaining studies were assessed through a full text review of the articles. The European Working Group on Sarcopenia (EWGS) and Asian Working Group for Sarcopenia (AWGS) were considered as the standard criteria for the diagnosis of sarcopenia (6, 7). After the systematic review, six articles were included (Table 1) (19–24). In all six articles, the AWGS criteria were used to diagnose sarcopenia (7).

**Table 1:** Characteristics of the included studies.

**No**.	**References**	**Country**	**Study design**	**No. of patients**	**Age (years)**	**Men: Women (*n*)**	**Sarcopenia/ Diabetes (*n*)**	**Sarcopenia/ non-diabetes (*n*)**	**Definition of sarcopenia**	**Body composition assessment**	**Participants recruitment method**	**Quality of evidence**
1	Han et al. (19)	China	Cross-sectional	1,069	≥60	467:602	21/139	78/930	AWGS	BIA	Residents from three areas (Hougu, East Chadian, and West Chadian) of Tianjin, China.	⊕⊕ (low)
2	Han et al. (20)	China	Cross-sectional	711	≥60	349:362	18/80	59/631	AWGS	BIA	Residents from three areas (Hougu, East Chadian, and West Chadian) of Tianjin, China.	⊕⊕ (low)
3	Kang et al. (21)	Korea	Cross-sectional	2,403	70–84	1135:1268	88/670	196/1733	AWGS	DXA	Part of Korean Frailty and Aging Cohort Study	⊕⊕ (low)
4	Keng et al. (22)	Singapore	Cross-sectional	378	≥65	197:181	26/75	62/303	AWGS	BIA	Part of Singapore Chinese Health study	⊕⊕ (low)
5	Nakamura et al. (23)	Japan	Cross-sectional	1,371	≥65	601:770	22/337	79/1034	AWGS	BIA	Part of Japan Hisayama Study	⊕⊕ (low)
6	Wang et al. (24)	China	Cross-sectional	1,090	60–95	520:570	35/236	96/854	AWGS	BIA	Community-dwelling citizens living in Zhenjiang city of Jiangsu province, China.	⊕⊕ (low)

### Study Characteristics

The selected studies included 1,537 diabetics and 5,485 non-diabetics. All patients were aged ≥60 years, and 53.4% were women. One study used dual-energy X-ray absorptiometry (DXA) to evaluate body composition, and another used bioelectrical impedance analysis (BIA).

### Risk of Bias

Three included studies [by Han et al. (19), Kang et al. (21), Nakamura et al. (23), and Wang et al. (24)] were rated nine stars (selection of subjects: fout stars; comparability of groups: two stars; and assessment of outcome: three stars). The remaining two studies [by Han et al. (20) and Keng et al. (22)] were rated eight stars (selection of subjects: four stars; comparability of groups: one star; and assessment of outcome: three stars). Therefore, the quality of all included studies assessed using NOS was considered high.

### Grading of Evidence

The overall quality (certainty) of evidence of all the included studies was rated as low.

### Meta-Analysis Results

**Figure 2:**
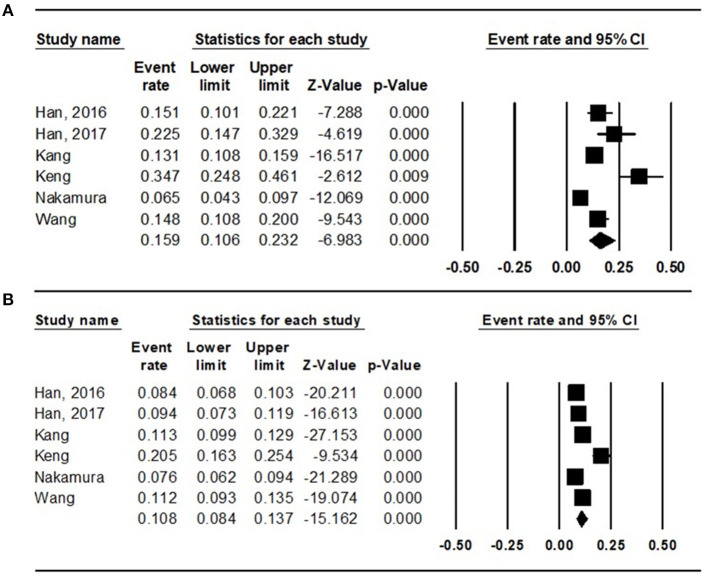
Results of the meta-analysis on sarcopenia prevalence among the **(A)** diabetes population and that among the **(B)** non-diabetes population.

To evaluate the prevalence of sarcopenia among the diabetic and non-diabetic population, the random effects model was used (diabetic: *I*^2^ = 88.472; non-diabetic: *I*^2^ = 89.158), and the total prevalence was 15.9% (95% CI = 10.6 to 23.2%) (Figure 2A) and 10.8% (95% CI = 8.4 to 13.7%) (Figure 2B), respectively.

**Figure 3:**
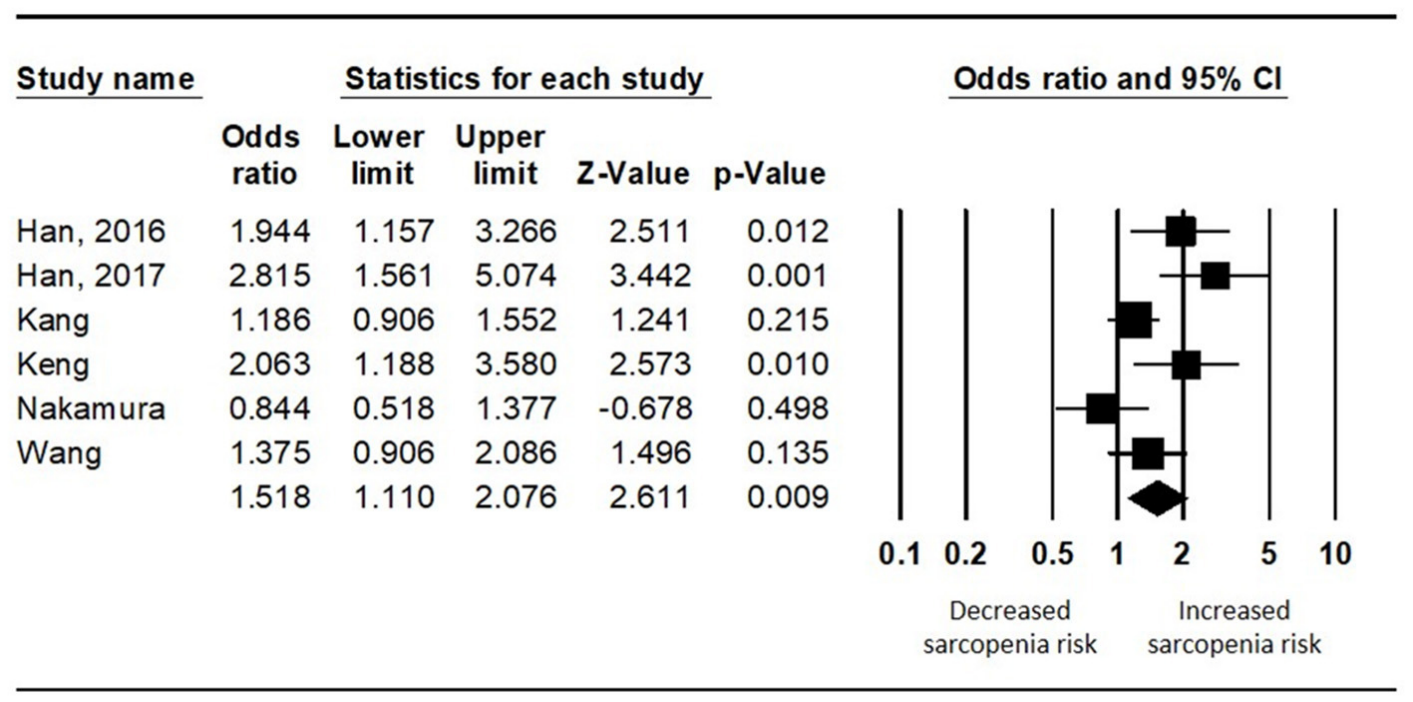
Results of the meta-analysis on the difference in the risk of sarcopenia between the diabetes population and the non-diabetes population.

To analyze the difference in the risk of sarcopenia between the diabetics and non-diabetics, the random effects model was used (*I*^2^ = 65.292). The diabetics showed a significantly higher risk of sarcopenia than the non-diabetics (pooled OR = 1.518, 95% CI = 1.110 to 2.076, Z-value = 2.611, *p* = 0.009) (Figure 3).

### Publication Bias

**Figure 4:**
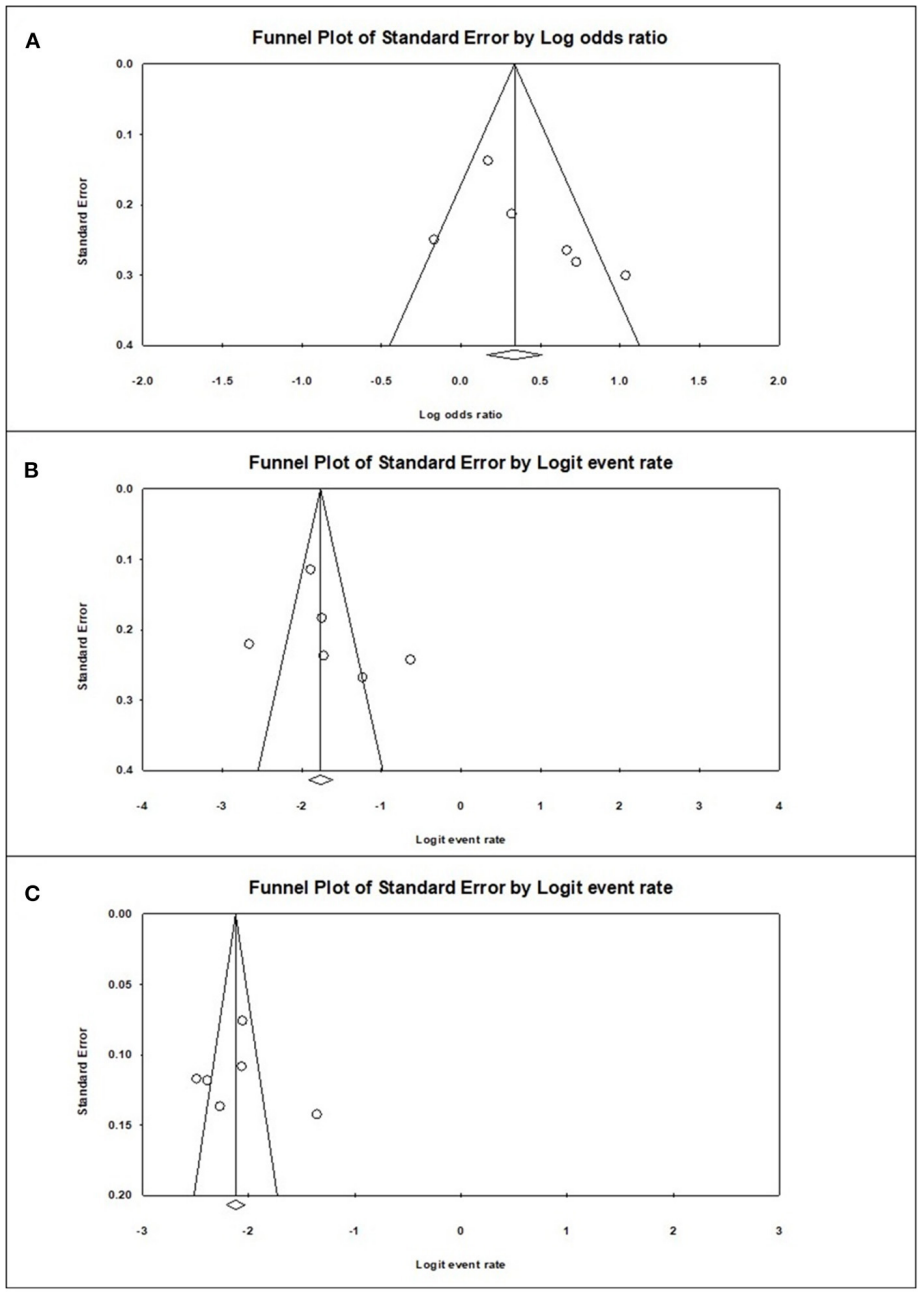
Graphic funnel plots of the included studies **(A)**, diabetes population vs. non-diabetic population; **(B)**, prevalence of sarcopenia among the diabetes population; **(C)**, prevalence of sarcopenia among the non-diabetic population.

Funnel plot analysis was performed, which showed visually a symmetrical distribution of published studies in all the analyses (prevalence of sarcopenia among the diabetics and non-diabetics and difference in the risk of sarcopenia between the diabetics and non-diabetics) (Figure 4).

## Discussion

In this meta-analysis, we analyzed studies that evaluated the prevalence of sarcopenia in diabetes and analyzed all the following parameters: muscle strength, quality, and function in the community-dwelling general population aged ≥ 60 years. During the systemic review, we found that most studies assessed either muscle strength or quality or did not target the general population. A total of six studies, which fulfilled all inclusion criteria were finally enrolled. The prevalence of sarcopenia was significantly higher in diabetics (15.9%) than non-diabetics (10.8%).

After the concept of “sarcopenia” was introduced by Baumgartner et al. (4), various definitions and diagnostic criteria have been introduced. The earlier diagnostic criteria were simply to assess skeletal muscle mass (25, 26), but recently, indicators to evaluate muscle function and quality are known to be considered an important portion of sarcopenia (5–7). Therefore, both components should be evaluated, and the diagnostic criteria reflecting them are widely accepted. Previous meta-analysis results included a number of studies using inconsistent criteria that demonstrated a wide range of the prevalence (2, 3). Since it is well-known that not only quantitative but also a qualitative assessment of body composition reflects the clinical outcomes such as frailty (21), fall risk (27), and mortality (28), we only included studies evaluated by EWGS or AWGS criteria recognized as the standard criteria (6, 7). Therefore, our findings have an advantage including the clinical studies conducted with more relevant criteria that are more closely related to actual clinical outcomes than previous studies. In addition, the risk of selection bias was less affected because only included the studies target the general population, not patients admitted to hospitals or patients receiving treatment for other diseases.

The evil synergistic effects between sarcopenia and metabolic disease (such as diabetes) are already established from large population studies which lead to critical outcomes such as cardiovascular disease or mortality. In a study on 6,021 (2,593 men, 3,429 women) individuals from the general population in South Korean aged 30–93 years, a low muscle mass increased the risk of metabolically unhealthy status (≥2 components of metabolic syndrome or the presence of hypertension, diabetes, or cardiovascular disease) in non-obese men by 1.88 folds (29). Of 347,130 individuals from the general population (158,959 men, 188,171 women; mean age, 55.9) in the United Kingdom, 4% were diabetic, and diabetics with low handgrip strength had a 4.05 times higher risk for cardiovascular disease mortality compared to the high handgrip strength group (30). Of 610 (diabetics, 306, non-diabetics, 304) patients who were hospitalized at a single center in Brazil, the risk of mortality after discharge was significantly higher in those with diabetes and sarcopenia (OR = 1.78) (28). Insulin resistance is known to be the one of major factors involving the development of sarcopenia (31, 32). In patients with diabetes, age-related anabolic resistance worsens, fat mass increases, and lean mass decreases more rapidly than in non-diabetics (33). Insulin plays a role in inhibiting protein catabolism in muscles, and insulin resistance induces protein dysregulation (31). Insulin resistance causes dysregulation of the potential mediators of glucose and protein metabolism in the skeletal muscles, which are as follows: those regulated by the hypothalamic-pituitary axis [glucocorticoids, insulin-like growth factor 1 (IGF-1), and androgen], as well as Akt/mammalian target of rapamycin signaling, AMP-activated protein kinase, myostatin, urocortins, and vitamin D (34). In addition, advanced glycation end-products accumulate in the skeletal muscles in chronic conditions, resulting in muscle mass reduction and sarcopenia (35). Tumor necrosis factor-α (TNF-α) and TNF-like weak inducer of apoptosis increase the risk of sarcopenia, while IGF-1, insulin, and adiponectin reduce the risk of sarcopenia (36). In elderly diabetes patients, the incidence of sarcopenic obesity (high body fat and low body mass) (37) should be evaluated and should be prevented by nutrition intake and exercise giving anabolic stimulus to skeletal muscle (38).

Among diabetic patients, there are differences in the risk of sarcopenia depending on the duration of diabetes and glucose variability. In a Japanese study on diabetic individuals aged ≥60 years (108 men and 105 women), the prevalence of sarcopenia was 19.2%. The risk of sarcopenia increased with increase in the duration of diabetes in women (OR = 1.43) (39). In another Japanese study on 746 diabetic patients aged 38–96 years, the prevalence of sarcopenia was 7%. The risk of sarcopenia was 7.2 times higher in the patients with glycated hemoglobin (HbA1c) ≥8% than that in patients with HbA1c <6.5% when blood glucose control was poor (40). In another study on Japanese diabetics aged 65–87 years (69 men, 33 women), the prevalence of sarcopenia was found to be 11.6%. Low muscle mass, low grip strength, and slow walking speed were associated with larger glucose fluctuations. Glucose fluctuation was reported to increase the risk of sarcopenia (OR = 1.045) (41). Our study assessed the association of diabetes and sarcopenia from the community-dwelling population but have some limitations regarding the effects of glycemic control status, disease duration, level of exercise, malnutrition, and hypoglycemic medications on sarcopenia in peoples with diabetes. Further large epidemiologic studies are warranted in the future. Interestingly, recent anti-diabetic therapeutics might be candidate for prevention of sarcopenia: the use of metformin, glucagon-like peptide-1(GLP-1) receptor agonists, and sodium-glucose co-transporter-2 (SGLT2) inhibitors, and metabolic surgery has been suggested as a potential strategy to reduce fat mass (33). Further prospective studies for the impact of glucose control and body composition modification by appropriate diabetes treatment strategy on sarcopenia prevention are needed.

A limitation of the current meta-analysis is that a relatively small number of relevant studies have been published that used standardized diagnostic criteria for sarcopenia in the community-dwelling general population. Despite this limitation, this systematic review retrieved comparatively large data on the difference in prevalence of sarcopenia among the diabetic and non-diabetic Asian populations.

In conclusion, this meta-analysis revealed that sarcopenia was more prevalent in the diabetic population than in the non-diabetic population. The presence of diabetes significantly affects the occurrence of sarcopenia. To reduce sarcopenia-related complications, proper nutrition intake and resistance exercises are essential. Especially in elderly individuals with diabetes, strategies to reduce glucose variability and maintain healthy body composition while managing cognitive dysfunction and accompanying cardiovascular risks are warranted.

## Data Availability Statement

The data analyzed in this study is subject to the following licenses/restrictions: this is a meta-analysis so the original dataset of reviewed studies is unavailable. Requests to access these datasets should be directed to Seung Min Chung, smchung@ynu.ac.kr.

## Author Contributions

SMC, JSM, and MCC: conception or design and drafting the work or revising. SMC and MCC: acquisition, analysis, or interpretation of data. All authors: final approval of the manuscript.

## Conflict of Interest

The authors declare that the research was conducted in the absence of any commercial or financial relationships that could be construed as a potential conflict of interest.
